# Bis(isopropoxido-κ*O*)bis­(2-methyl­quinolin-8-olato-κ^2^ *N*,*O*)titanium(IV)

**DOI:** 10.1107/S1600536808035460

**Published:** 2008-11-08

**Authors:** Yousef Fazaeli, Mostafa M. Amini, Seik Weng Ng

**Affiliations:** a Department of Chemistry, Shahid Beheshti University, Tehran, Iran; b Department of Chemistry, University of Malaya, 50603 Kuala Lumpur, Malaysia

## Abstract

The two 2-methyl­quinolin-8-olate anions in the title complex, [Ti(C_10_H_8_NO)_2_(C_3_H_7_O)_2_], chelate the Ti^IV^ atom, which shows an all-*cis* distorted octa­hedral N_2_O_4_ coordination geometry.

## Related literature

For the synthesis, see: Bickley & Nick (1979 ▶); Harrod & Taylor (1975 ▶). For the crystal structure of bis­(isoprop­oxy)bis(quinolin-8-olato)titanium, see: Zeng *et al.* (2002 ▶).
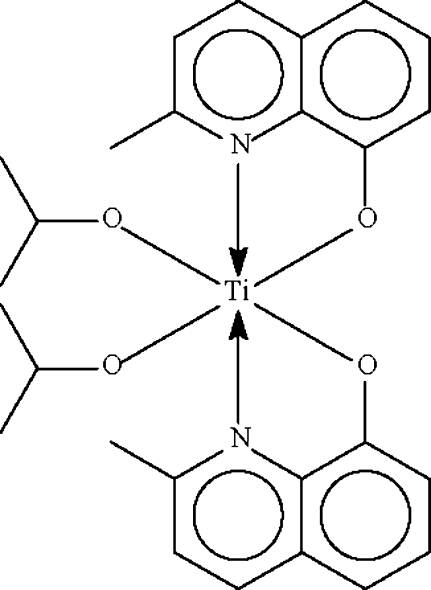


## Experimental

### 

#### Crystal data

[Ti(C_10_H_8_NO)_2_(C_3_H_7_O)_2_]
*M* *_r_* = 482.42
Monoclinic, 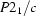

*a* = 9.5851 (2) Å
*b* = 13.5768 (2) Å
*c* = 18.7779 (3) Å
β = 102.559 (1)°
*V* = 2385.19 (7) Å^3^
*Z* = 4
Mo *K*α radiation
μ = 0.39 mm^−1^
*T* = 100 (2) K
0.35 × 0.25 × 0.15 mm

#### Data collection

Bruker SMART APEX diffractometer
Absorption correction: multi-scan (*SADABS*; Sheldrick, 1996 ▶) *T*_min_ = 0.875, *T*_max_ = 0.943
16391 measured reflections
5467 independent reflections
4651 reflections with *I* > 2σ(*I*)
*R*_int_ = 0.025

#### Refinement

*R*[*F* ^2^ > 2σ(*F* ^2^)] = 0.032
*wR*(*F* ^2^) = 0.088
*S* = 1.03
5467 reflections
304 parameters
H-atom parameters constrained
Δρ_max_ = 0.39 e Å^−3^
Δρ_min_ = −0.39 e Å^−3^

### 

Data collection: *APEX2* (Bruker, 2007 ▶); cell refinement: *APEX2*; data reduction: *SAINT* (Bruker, 2007 ▶); program(s) used to solve structure: *SHELXS97* (Sheldrick, 2008 ▶); program(s) used to refine structure: *SHELXL97* (Sheldrick, 2008 ▶); molecular graphics: *X-SEED* (Barbour, 2001 ▶); software used to prepare material for publication: *publCIF* (Westrip, 2008 ▶).

## Supplementary Material

Crystal structure: contains datablocks global, I. DOI: 10.1107/S1600536808035460/tk2320sup1.cif

Structure factors: contains datablocks I. DOI: 10.1107/S1600536808035460/tk2320Isup2.hkl

Additional supplementary materials:  crystallographic information; 3D view; checkCIF report

## Figures and Tables

**Fig. 1.**
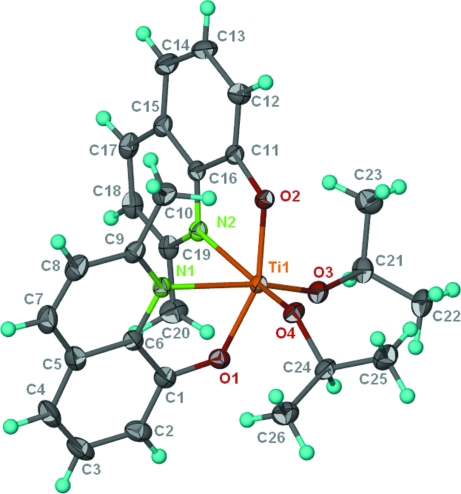
Thermal ellipsoid plot (Barbour, 2001) of Ti(C10H8NO)2(C3H7O)2 at the 70% probability level showing atom labelling. Hydrogen atoms are drawn as spheres of arbitrary radius.

## supplementary crystallographic information

### Experimental

8-Hydroxy-2-methylquinoline (1.59 g, 10 mmol) was added to the titanium isopropoxide (2.84 g, 10 mmol) in toluene (20 ml) at room temperature. The mixture was stirred for a day and than solvent was removed under reduced pressure to furnish an orange solid. The solid was crystallized from dichloromethane and *n*-hexane (1:1) to give yellow crystals, m.p. 445 K. IR (KBr, cm^-1^): 1575 (C═C, C═N), 1236 (C—O). ^1^H NMR (CDCl_3_, p.p.m.): 0.94 (CH_3_, doublet), 1.14 (CH_3_, doublet), 2.83 (CH_3_, singlet), 4.61 (CH, quartet), 6.9–8.58 (aromatic H atoms).

### Refinement

Hydrogen atoms were placed in their calculated positions (C—H 0.95–0.98 Å) and were treated as riding on their parent atoms, with *U*(H) set to 1.2–1.5 times *U*_eq_(C).

### Crystal data

**Table:**

[Ti(C_10_H_8_NO)_2_(C_3_H_7_O)_2_]	*F*_000_ = 1016
*M**_r_* = 482.42	*D*_x_ = 1.343 Mg m^−^^3^
Monoclinic, *P*2_1_/*c*	Mo *K*α radiation λ = 0.71073 Å
Hall symbol: -P 2ybc	Cell parameters from 6457 reflections
*a* = 9.5851 (2) Å	θ = 2.2–28.3º
*b* = 13.5768 (2) Å	µ = 0.39 mm^−^^1^
*c* = 18.7779 (3) Å	*T* = 100 (2) K
β = 102.559 (1)º	Irregular chip, yellow
*V* = 2385.19 (7) Å^3^	0.35 × 0.25 × 0.15 mm
*Z* = 4	

### Data collection

**Table:**

Bruker SMART APEX diffractometer	5467 independent reflections
Radiation source: fine-focus sealed tube	4651 reflections with *I* > 2σ(*I*)
Monochromator: graphite	*R*_int_ = 0.025
*T* = 100(2) K	θ_max_ = 27.5º
ω scans	θ_min_ = 2.2º
Absorption correction: multi-scan(SADABS; Sheldrick, 1996)	*h* = −12→11
*T*_min_ = 0.875, *T*_max_ = 0.943	*k* = −17→17
16391 measured reflections	*l* = −24→24

### Refinement

**Table:**

Refinement on *F*^2^	Secondary atom site location: difference Fourier map
Least-squares matrix: full	Hydrogen site location: inferred from neighbouring sites
*R*[*F*^2^ > 2σ(*F*^2^)] = 0.032	H-atom parameters constrained
*wR*(*F*^2^) = 0.088	*w* = 1/[σ^2^(*F*_o_^2^) + (0.0394*P*)^2^ + 1.3426*P*] where *P* = (*F*_o_^2^ + 2*F*_c_^2^)/3
*S* = 1.03	(Δ/σ)_max_ = 0.001
5467 reflections	Δρ_max_ = 0.39 e Å^−^^3^
304 parameters	Δρ_min_ = −0.39 e Å^−^^3^
Primary atom site location: structure-invariant direct methods	Extinction correction: none

### Special details

**Table:**

Geometry. All esds (except the esd in the dihedral angle between two l.s. planes) are estimated using the full covariance matrix. The cell esds are taken into account individually in the estimation of esds in distances, angles and torsion angles; correlations between esds in cell parameters are only used when they are defined by crystal symmetry. An approximate (isotropic) treatment of cell esds is used for estimating esds involving l.s. planes.

### Fractional atomic coordinates and isotropic or equivalent isotropic displacement parameters (Å^2^)

**Table:**

	*x*	*y*	*z*	*U*_iso_*/*U*_eq_	
Ti1	0.36712 (3)	0.433571 (18)	0.241664 (14)	0.01288 (8)	
O1	0.26537 (11)	0.55637 (7)	0.23824 (6)	0.0166 (2)	
O2	0.46090 (11)	0.31617 (7)	0.28355 (5)	0.0153 (2)	
O3	0.49414 (11)	0.48288 (8)	0.19514 (6)	0.0187 (2)	
O4	0.24248 (11)	0.37144 (8)	0.16802 (5)	0.0171 (2)	
N1	0.19366 (13)	0.41035 (9)	0.31601 (6)	0.0142 (2)	
N2	0.51257 (13)	0.48222 (9)	0.35581 (6)	0.0145 (2)	
C1	0.16171 (16)	0.57482 (11)	0.27306 (8)	0.0169 (3)	
C2	0.09311 (17)	0.66473 (12)	0.27024 (9)	0.0219 (3)	
H2	0.1206	0.7172	0.2427	0.026*	
C3	−0.01715 (18)	0.67913 (12)	0.30789 (9)	0.0261 (4)	
H3	−0.0628	0.7415	0.3055	0.031*	
C4	−0.05984 (17)	0.60535 (13)	0.34771 (9)	0.0259 (4)	
H4	−0.1351	0.6164	0.3724	0.031*	
C5	0.00847 (16)	0.51221 (12)	0.35212 (8)	0.0204 (3)	
C6	0.12004 (15)	0.49752 (11)	0.31484 (8)	0.0155 (3)	
C7	−0.02862 (17)	0.43106 (13)	0.39098 (9)	0.0244 (3)	
H7	−0.1022	0.4370	0.4174	0.029*	
C8	0.04179 (17)	0.34421 (13)	0.39039 (9)	0.0228 (3)	
H8	0.0155	0.2890	0.4157	0.027*	
C9	0.15426 (16)	0.33490 (11)	0.35243 (8)	0.0170 (3)	
C10	0.22910 (17)	0.23783 (11)	0.35446 (9)	0.0210 (3)	
H10A	0.2668	0.2303	0.3102	0.032*	
H10B	0.1615	0.1845	0.3570	0.032*	
H10C	0.3081	0.2352	0.3975	0.032*	
C11	0.54608 (15)	0.31020 (11)	0.34980 (8)	0.0151 (3)	
C12	0.60985 (17)	0.22382 (12)	0.37911 (8)	0.0197 (3)	
H12	0.5961	0.1647	0.3514	0.024*	
C13	0.69565 (17)	0.22374 (12)	0.45060 (9)	0.0226 (3)	
H13	0.7381	0.1638	0.4706	0.027*	
C14	0.71930 (17)	0.30763 (12)	0.49184 (9)	0.0215 (3)	
H14	0.7760	0.3054	0.5401	0.026*	
C15	0.65868 (16)	0.39776 (12)	0.46212 (8)	0.0170 (3)	
C16	0.57156 (15)	0.39851 (11)	0.39100 (8)	0.0145 (3)	
C17	0.68312 (16)	0.49006 (12)	0.49736 (8)	0.0199 (3)	
H17	0.7367	0.4940	0.5462	0.024*	
C18	0.62969 (17)	0.57309 (11)	0.46108 (8)	0.0197 (3)	
H18	0.6493	0.6353	0.4842	0.024*	
C19	0.54487 (16)	0.56820 (11)	0.38889 (8)	0.0171 (3)	
C20	0.49429 (18)	0.66222 (11)	0.35024 (9)	0.0235 (3)	
H20A	0.4595	0.6491	0.2981	0.035*	
H20B	0.5736	0.7093	0.3570	0.035*	
H20C	0.4166	0.6899	0.3704	0.035*	
C21	0.63093 (17)	0.48966 (12)	0.17852 (9)	0.0199 (3)	
H21	0.6754	0.5535	0.1980	0.024*	
C22	0.6130 (2)	0.48986 (15)	0.09632 (9)	0.0321 (4)	
H22A	0.5505	0.5445	0.0754	0.048*	
H22B	0.5703	0.4274	0.0764	0.048*	
H22C	0.7066	0.4978	0.0840	0.048*	
C23	0.72535 (18)	0.40664 (13)	0.21459 (10)	0.0274 (4)	
H23A	0.7328	0.4091	0.2674	0.041*	
H23B	0.8207	0.4135	0.2042	0.041*	
H23C	0.6838	0.3434	0.1956	0.041*	
C24	0.17566 (17)	0.41249 (11)	0.09929 (8)	0.0181 (3)	
H24	0.2249	0.4755	0.0922	0.022*	
C25	0.1915 (2)	0.34129 (13)	0.03960 (9)	0.0308 (4)	
H25A	0.2932	0.3306	0.0411	0.046*	
H25B	0.1451	0.3687	−0.0079	0.046*	
H25C	0.1464	0.2784	0.0470	0.046*	
C26	0.02027 (18)	0.43437 (14)	0.09889 (10)	0.0279 (4)	
H26A	0.0150	0.4809	0.1381	0.042*	
H26B	−0.0286	0.3731	0.1065	0.042*	
H26C	−0.0259	0.4632	0.0518	0.042*	

### Atomic displacement parameters (Å^2^)

**Table:**

	*U*^11^	*U*^22^	*U*^33^	*U*^12^	*U*^13^	*U*^23^
Ti1	0.01437 (14)	0.01316 (13)	0.01128 (13)	0.00042 (10)	0.00319 (9)	0.00035 (9)
O1	0.0175 (5)	0.0147 (5)	0.0178 (5)	0.0012 (4)	0.0042 (4)	0.0013 (4)
O2	0.0170 (5)	0.0156 (5)	0.0125 (5)	0.0015 (4)	0.0016 (4)	−0.0008 (4)
O3	0.0177 (5)	0.0219 (5)	0.0178 (5)	0.0005 (4)	0.0068 (4)	0.0032 (4)
O4	0.0201 (5)	0.0170 (5)	0.0130 (5)	−0.0005 (4)	0.0013 (4)	0.0005 (4)
N1	0.0133 (6)	0.0169 (6)	0.0119 (6)	−0.0007 (5)	0.0017 (5)	−0.0018 (4)
N2	0.0132 (6)	0.0164 (6)	0.0144 (6)	−0.0013 (5)	0.0041 (5)	0.0001 (5)
C1	0.0155 (7)	0.0176 (7)	0.0158 (7)	−0.0004 (6)	−0.0006 (5)	−0.0037 (5)
C2	0.0216 (8)	0.0172 (7)	0.0240 (8)	0.0025 (6)	−0.0016 (6)	−0.0030 (6)
C3	0.0224 (8)	0.0231 (8)	0.0296 (9)	0.0087 (7)	−0.0013 (7)	−0.0095 (7)
C4	0.0170 (8)	0.0343 (9)	0.0262 (8)	0.0060 (7)	0.0039 (6)	−0.0099 (7)
C5	0.0136 (7)	0.0289 (8)	0.0177 (7)	0.0009 (6)	0.0011 (6)	−0.0054 (6)
C6	0.0131 (7)	0.0185 (7)	0.0133 (7)	0.0000 (6)	−0.0005 (5)	−0.0034 (5)
C7	0.0162 (8)	0.0388 (10)	0.0197 (8)	−0.0011 (7)	0.0071 (6)	−0.0023 (7)
C8	0.0192 (8)	0.0305 (9)	0.0189 (8)	−0.0048 (7)	0.0049 (6)	0.0035 (6)
C9	0.0154 (7)	0.0214 (7)	0.0129 (7)	−0.0034 (6)	0.0003 (5)	0.0001 (5)
C10	0.0216 (8)	0.0205 (8)	0.0214 (8)	−0.0019 (6)	0.0056 (6)	0.0045 (6)
C11	0.0128 (7)	0.0189 (7)	0.0139 (7)	0.0002 (5)	0.0034 (5)	0.0007 (5)
C12	0.0196 (8)	0.0188 (7)	0.0205 (8)	0.0022 (6)	0.0042 (6)	0.0016 (6)
C13	0.0208 (8)	0.0237 (8)	0.0223 (8)	0.0055 (6)	0.0025 (6)	0.0077 (6)
C14	0.0173 (8)	0.0296 (8)	0.0164 (7)	0.0009 (6)	0.0010 (6)	0.0047 (6)
C15	0.0136 (7)	0.0240 (8)	0.0140 (7)	−0.0016 (6)	0.0042 (6)	0.0013 (6)
C16	0.0122 (7)	0.0182 (7)	0.0141 (7)	−0.0002 (5)	0.0049 (5)	0.0014 (5)
C17	0.0152 (7)	0.0301 (8)	0.0142 (7)	−0.0047 (6)	0.0030 (6)	−0.0032 (6)
C18	0.0183 (8)	0.0221 (8)	0.0191 (7)	−0.0062 (6)	0.0052 (6)	−0.0061 (6)
C19	0.0151 (7)	0.0188 (7)	0.0182 (7)	−0.0037 (6)	0.0057 (6)	−0.0018 (6)
C20	0.0278 (9)	0.0167 (7)	0.0238 (8)	−0.0025 (6)	0.0009 (7)	−0.0008 (6)
C21	0.0189 (8)	0.0216 (8)	0.0210 (8)	−0.0041 (6)	0.0081 (6)	−0.0003 (6)
C22	0.0325 (10)	0.0447 (11)	0.0233 (9)	0.0086 (8)	0.0156 (7)	0.0071 (8)
C23	0.0180 (8)	0.0340 (9)	0.0305 (9)	0.0017 (7)	0.0059 (7)	0.0053 (7)
C24	0.0218 (8)	0.0189 (7)	0.0130 (7)	0.0004 (6)	0.0024 (6)	0.0019 (5)
C25	0.0447 (11)	0.0302 (9)	0.0160 (8)	0.0091 (8)	0.0031 (7)	−0.0011 (7)
C26	0.0225 (9)	0.0362 (10)	0.0245 (8)	0.0057 (7)	0.0037 (7)	0.0071 (7)

### Geometric parameters (Å, °)

**Table:**

Ti1—O3	1.7766 (11)	C12—C13	1.414 (2)
Ti1—O4	1.8255 (10)	C12—H12	0.9500
Ti1—O2	1.9130 (10)	C13—C14	1.368 (2)
Ti1—O1	1.9255 (10)	C13—H13	0.9500
Ti1—N2	2.3822 (12)	C14—C15	1.416 (2)
Ti1—N1	2.4130 (12)	C14—H14	0.9500
O1—C1	1.3266 (18)	C15—C16	1.413 (2)
O2—C11	1.3338 (17)	C15—C17	1.413 (2)
O3—C21	1.4153 (18)	C17—C18	1.359 (2)
O4—C24	1.4239 (17)	C17—H17	0.9500
N1—C9	1.3311 (19)	C18—C19	1.422 (2)
N1—C6	1.3756 (19)	C18—H18	0.9500
N2—C19	1.3270 (19)	C19—C20	1.496 (2)
N2—C16	1.3731 (19)	C20—H20A	0.9800
C1—C2	1.382 (2)	C20—H20B	0.9800
C1—C6	1.419 (2)	C20—H20C	0.9800
C2—C3	1.407 (2)	C21—C23	1.511 (2)
C2—H2	0.9500	C21—C22	1.515 (2)
C3—C4	1.365 (3)	C21—H21	1.0000
C3—H3	0.9500	C22—H22A	0.9800
C4—C5	1.418 (2)	C22—H22B	0.9800
C4—H4	0.9500	C22—H22C	0.9800
C5—C7	1.409 (2)	C23—H23A	0.9800
C5—C6	1.414 (2)	C23—H23B	0.9800
C7—C8	1.360 (2)	C23—H23C	0.9800
C7—H7	0.9500	C24—C25	1.512 (2)
C8—C9	1.421 (2)	C24—C26	1.517 (2)
C8—H8	0.9500	C24—H24	1.0000
C9—C10	1.497 (2)	C25—H25A	0.9800
C10—H10A	0.9800	C25—H25B	0.9800
C10—H10B	0.9800	C25—H25C	0.9800
C10—H10C	0.9800	C26—H26A	0.9800
C11—C12	1.380 (2)	C26—H26B	0.9800
C11—C16	1.419 (2)	C26—H26C	0.9800
			
O3—Ti1—O4	101.96 (5)	C14—C13—C12	121.83 (15)
O3—Ti1—O2	101.84 (5)	C14—C13—H13	119.1
O4—Ti1—O2	95.62 (4)	C12—C13—H13	119.1
O3—Ti1—O1	93.07 (5)	C13—C14—C15	119.54 (14)
O4—Ti1—O1	97.56 (5)	C13—C14—H14	120.2
O2—Ti1—O1	157.58 (4)	C15—C14—H14	120.2
O3—Ti1—N2	90.48 (5)	C16—C15—C17	116.11 (14)
O4—Ti1—N2	165.52 (4)	C16—C15—C14	119.09 (14)
O2—Ti1—N2	74.36 (4)	C17—C15—C14	124.74 (14)
O1—Ti1—N2	89.03 (4)	N2—C16—C15	123.90 (14)
O3—Ti1—N1	164.98 (5)	N2—C16—C11	115.65 (13)
O4—Ti1—N1	87.60 (4)	C15—C16—C11	120.37 (14)
O2—Ti1—N1	88.54 (4)	C18—C17—C15	119.67 (14)
O1—Ti1—N1	74.02 (4)	C18—C17—H17	120.2
N2—Ti1—N1	81.82 (4)	C15—C17—H17	120.2
C1—O1—Ti1	125.12 (9)	C17—C18—C19	120.95 (14)
C11—O2—Ti1	124.87 (9)	C17—C18—H18	119.5
C21—O3—Ti1	154.13 (10)	C19—C18—H18	119.5
C24—O4—Ti1	126.66 (9)	N2—C19—C18	120.93 (14)
C9—N1—C6	117.94 (13)	N2—C19—C20	120.36 (14)
C9—N1—Ti1	134.90 (10)	C18—C19—C20	118.70 (13)
C6—N1—Ti1	107.09 (9)	C19—C20—H20A	109.5
C19—N2—C16	118.24 (13)	C19—C20—H20B	109.5
C19—N2—Ti1	134.14 (10)	H20A—C20—H20B	109.5
C16—N2—Ti1	107.62 (9)	C19—C20—H20C	109.5
O1—C1—C2	123.29 (14)	H20A—C20—H20C	109.5
O1—C1—C6	117.65 (13)	H20B—C20—H20C	109.5
C2—C1—C6	119.06 (14)	O3—C21—C23	110.24 (12)
C1—C2—C3	120.43 (15)	O3—C21—C22	108.63 (13)
C1—C2—H2	119.8	C23—C21—C22	112.47 (14)
C3—C2—H2	119.8	O3—C21—H21	108.5
C4—C3—C2	121.38 (15)	C23—C21—H21	108.5
C4—C3—H3	119.3	C22—C21—H21	108.5
C2—C3—H3	119.3	C21—C22—H22A	109.5
C3—C4—C5	119.79 (15)	C21—C22—H22B	109.5
C3—C4—H4	120.1	H22A—C22—H22B	109.5
C5—C4—H4	120.1	C21—C22—H22C	109.5
C7—C5—C6	116.69 (14)	H22A—C22—H22C	109.5
C7—C5—C4	124.21 (15)	H22B—C22—H22C	109.5
C6—C5—C4	119.09 (15)	C21—C23—H23A	109.5
N1—C6—C5	123.64 (14)	C21—C23—H23B	109.5
N1—C6—C1	116.11 (13)	H23A—C23—H23B	109.5
C5—C6—C1	120.24 (14)	C21—C23—H23C	109.5
C8—C7—C5	119.49 (15)	H23A—C23—H23C	109.5
C8—C7—H7	120.3	H23B—C23—H23C	109.5
C5—C7—H7	120.3	O4—C24—C25	108.89 (12)
C7—C8—C9	120.86 (15)	O4—C24—C26	109.19 (12)
C7—C8—H8	119.6	C25—C24—C26	112.20 (14)
C9—C8—H8	119.6	O4—C24—H24	108.8
N1—C9—C8	121.34 (14)	C25—C24—H24	108.8
N1—C9—C10	120.18 (13)	C26—C24—H24	108.8
C8—C9—C10	118.48 (13)	C24—C25—H25A	109.5
C9—C10—H10A	109.5	C24—C25—H25B	109.5
C9—C10—H10B	109.5	H25A—C25—H25B	109.5
H10A—C10—H10B	109.5	C24—C25—H25C	109.5
C9—C10—H10C	109.5	H25A—C25—H25C	109.5
H10A—C10—H10C	109.5	H25B—C25—H25C	109.5
H10B—C10—H10C	109.5	C24—C26—H26A	109.5
O2—C11—C12	123.48 (13)	C24—C26—H26B	109.5
O2—C11—C16	117.07 (13)	H26A—C26—H26B	109.5
C12—C11—C16	119.45 (13)	C24—C26—H26C	109.5
C11—C12—C13	119.68 (15)	H26A—C26—H26C	109.5
C11—C12—H12	120.2	H26B—C26—H26C	109.5
C13—C12—H12	120.2		
			
O3—Ti1—O1—C1	−171.18 (11)	Ti1—N1—C6—C1	−0.15 (14)
O4—Ti1—O1—C1	86.32 (11)	C7—C5—C6—N1	−1.2 (2)
O2—Ti1—O1—C1	−39.19 (19)	C4—C5—C6—N1	179.55 (14)
N2—Ti1—O1—C1	−80.75 (11)	C7—C5—C6—C1	178.57 (14)
N1—Ti1—O1—C1	1.02 (11)	C4—C5—C6—C1	−0.6 (2)
O3—Ti1—O2—C11	91.30 (11)	O1—C1—C6—N1	0.93 (19)
O4—Ti1—O2—C11	−165.20 (11)	C2—C1—C6—N1	−179.29 (13)
O1—Ti1—O2—C11	−39.37 (18)	O1—C1—C6—C5	−178.89 (13)
N2—Ti1—O2—C11	4.16 (10)	C2—C1—C6—C5	0.9 (2)
N1—Ti1—O2—C11	−77.75 (11)	C6—C5—C7—C8	−0.6 (2)
O4—Ti1—O3—C21	−112.2 (2)	C4—C5—C7—C8	178.58 (15)
O2—Ti1—O3—C21	−13.8 (2)	C5—C7—C8—C9	1.4 (2)
O1—Ti1—O3—C21	149.4 (2)	C6—N1—C9—C8	−1.4 (2)
N2—Ti1—O3—C21	60.3 (2)	Ti1—N1—C9—C8	−177.92 (10)
N1—Ti1—O3—C21	119.1 (2)	C6—N1—C9—C10	178.99 (13)
O3—Ti1—O4—C24	−50.83 (12)	Ti1—N1—C9—C10	2.4 (2)
O2—Ti1—O4—C24	−154.22 (11)	C7—C8—C9—N1	−0.4 (2)
O1—Ti1—O4—C24	43.96 (12)	C7—C8—C9—C10	179.26 (15)
N2—Ti1—O4—C24	160.41 (16)	Ti1—O2—C11—C12	178.57 (11)
N1—Ti1—O4—C24	117.49 (11)	Ti1—O2—C11—C16	−1.88 (18)
O3—Ti1—N1—C9	−152.04 (17)	O2—C11—C12—C13	−178.34 (14)
O4—Ti1—N1—C9	77.85 (14)	C16—C11—C12—C13	2.1 (2)
O2—Ti1—N1—C9	−17.84 (14)	C11—C12—C13—C14	−0.8 (2)
O1—Ti1—N1—C9	176.41 (14)	C12—C13—C14—C15	−1.1 (2)
N2—Ti1—N1—C9	−92.25 (14)	C13—C14—C15—C16	1.7 (2)
O3—Ti1—N1—C6	31.1 (2)	C13—C14—C15—C17	−175.58 (15)
O4—Ti1—N1—C6	−98.96 (9)	C19—N2—C16—C15	3.5 (2)
O2—Ti1—N1—C6	165.34 (9)	Ti1—N2—C16—C15	−176.65 (11)
O1—Ti1—N1—C6	−0.40 (9)	C19—N2—C16—C11	−173.27 (13)
N2—Ti1—N1—C6	90.94 (9)	Ti1—N2—C16—C11	6.53 (14)
O3—Ti1—N2—C19	71.95 (14)	C17—C15—C16—N2	0.4 (2)
O4—Ti1—N2—C19	−138.53 (18)	C14—C15—C16—N2	−177.12 (13)
O2—Ti1—N2—C19	174.12 (14)	C17—C15—C16—C11	177.10 (13)
O1—Ti1—N2—C19	−21.11 (14)	C14—C15—C16—C11	−0.4 (2)
N1—Ti1—N2—C19	−95.11 (14)	O2—C11—C16—N2	−4.13 (19)
O3—Ti1—N2—C16	−107.81 (9)	C12—C11—C16—N2	175.45 (13)
O4—Ti1—N2—C16	41.7 (2)	O2—C11—C16—C15	178.93 (12)
O2—Ti1—N2—C16	−5.64 (9)	C12—C11—C16—C15	−1.5 (2)
O1—Ti1—N2—C16	159.13 (9)	C16—C15—C17—C18	−3.3 (2)
N1—Ti1—N2—C16	85.13 (9)	C14—C15—C17—C18	174.09 (15)
Ti1—O1—C1—C2	178.74 (11)	C15—C17—C18—C19	2.3 (2)
Ti1—O1—C1—C6	−1.49 (18)	C16—N2—C19—C18	−4.6 (2)
O1—C1—C2—C3	179.34 (14)	Ti1—N2—C19—C18	175.66 (10)
C6—C1—C2—C3	−0.4 (2)	C16—N2—C19—C20	174.44 (13)
C1—C2—C3—C4	−0.3 (2)	Ti1—N2—C19—C20	−5.3 (2)
C2—C3—C4—C5	0.6 (2)	C17—C18—C19—N2	1.8 (2)
C3—C4—C5—C7	−179.23 (16)	C17—C18—C19—C20	−177.27 (14)
C3—C4—C5—C6	−0.1 (2)	Ti1—O3—C21—C23	8.2 (3)
C9—N1—C6—C5	2.2 (2)	Ti1—O3—C21—C22	131.9 (2)
Ti1—N1—C6—C5	179.66 (12)	Ti1—O4—C24—C25	132.10 (12)
C9—N1—C6—C1	−177.60 (13)	Ti1—O4—C24—C26	−105.08 (14)
